# Forecasting methodology with structural auto-adaptive intelligent grey models

**DOI:** 10.1016/j.mex.2023.102237

**Published:** 2023-06-02

**Authors:** Flavian Emmanuel Sapnken, Jean Gaston Tamba

**Affiliations:** a Laboratory of Technologies and Applied Science, PO Box 8698, IUT Douala, Douala, Cameroon; b Applied Logistics and Transport Laboratory, PO Box 8698, IUT Douala, Douala, Cameroon

**Keywords:** Grey forecasting model, Structural flexibility, Parameterization, Modelling knowledge, SAIGM model, Structural auto-adaptive intelligent grey model (SAIGM)

## Abstract

Accurate mid- and long-term petroleum products (PP) consumption forecasting is vital for strategic reserve management and energy planning. In order to address the issue of energy forecasting, a novel structural auto-adaptive intelligent grey model (SAIGM) is developed in this paper. To start with, a novel time response function for predictions that corrects the main weaknesses of the traditional grey model is established. Then, the optimal parameter values are calculated using SAIGM to increase adaptability and flexibility to deal with a variety of forecasting dilemmas. The viability and performance of SAIGM are examined with both ideal and real-world data. The former is constructed from algebraic series while the latter is made up Cameroon's PP consumption data. With its ingrained structural flexibility, SAIGM yields forecasts with RMSE of 3.10 and 1.54% MAPE. The proposed model performs better than competing intelligent grey systems that have been developed to date and is thus a valid forecasting tool that can be used to track the growth of Cameroon's PP demand.

• The ability of SAIGM enhances the forecasting power of intelligent grey models to fully extracting the laws of a system, no matter the data specifications.
• SAIGM is extended to include quasi-exponential series by addressing structural flexibility and parametrization concerns.
• Input attributes determination and data preprocessing are not required for the proposed model.

## Graphical abstract

**Figure:**
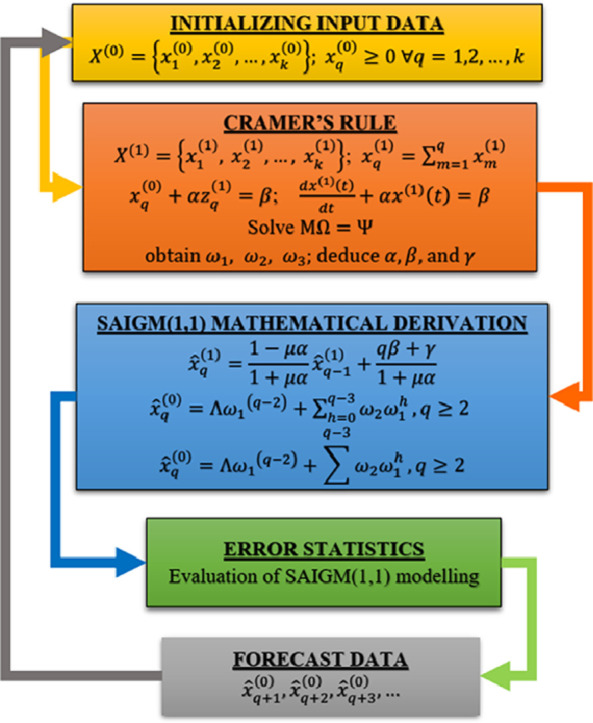

Specifications table

**Table:** 

Subject area:	Energy
More specific subject area:	*Modeling and forecasting*
Name of your method:	*Structural auto-adaptive intelligent grey model (SAIGM)*
Name and reference of original method	*J. Cui, Y.G. Dang, S.F. Liu, Novel grey forecasting model and its modeling mechanism, Control and Decision. 24 (2009) 1702–1706.* *N.-M. Xie, S.-F. Liu, Y.-J. Yang, C.-Q. Yuan, On novel grey forecasting model based on non-homogeneous index sequence, Applied Mathematical Modelling. 37 (2013) 5059–5068.* *W. Zhou, J.-M. He, Generalized GM (1,1) model and its application in forecasting of fuel production, Applied Mathematical Modelling. 37 (2013) 6234–6243.**https://doi.org/10.1016/j.apm.2013.01.002*.
Resource availability	*Petroleum Products data (**https://www.scdp.cm**)*

## Introduction

### Interest of SAIGM

Predictive outcomes for series with a homogeneous uniform exponential law are good when the conventional grey model (GM) is used [1], but this is not the case for data with anomalous properties, such as volatilities, periodicities and sinusoidal trends [2]. Numerous works have suggested enhanced GMs, including trigonometric (sinωt-GM), discrete (DGM) and seasonal (SGM) models [3,4]. Unfortunately, it is challenging to capture non-linear variations in some series due to the rigid structure of such systems. Therefore, it is crucial to figure out how to choose a structure that is suitable for the actual properties of the data. Another problem that requires attention is the GM's temporal reaction function.

As a result, this study develops a structural intelligent generalized model (SAIGM) that automatically adjusts to data features and completely captures a system's evolution, no matter its heterogeneity, homogeneity, volatility, linearity and periodicity. The parameters of SAIGM and the temporal response functions are calculated in this paper using differential equations. By using this method, SAIGM is relieved of the burden of dealing with parametrization concerns and produces quick simulations and exceptionally accurate forecasts.

### Contributions and novelty

Qian and Sui [5] as well as Sapnken and Tamba [6] demonstrate that GMs yield precise energy forecasts, however they are still flawed when the input data are characterized by trends other than exponential growths. In the case of a seasonality index, SGM(1,1) can only represent simple periodic variations. Discrete models can capture various types of variation, but SGM cannot. Nonetheless, discrete GMs have a jump flaw in their temporal response function that results in information loss [5]. Last but not least, the design of data pretreatment models makes it difficult for them to flexibly adjust to the nonlinearities ingrained in time series, lowering precision at each forecasting period. This work creates a structural self-adaptive intelligent GM (SAIGM(1,1)) to address these limitations. This method thus offers three contributions:

• SAIGM(1,1) is developed to enhance the prediction capabilities of intelligent GMs by enabling it to completely explore a system's laws of evolution, no matter the properties of time series used.
• SAIGM(1,1) applies to random, linear, and quasi-exponential series in addition to pure exponential series because it addresses the structural flexibility and parametrization concerns.
• SAIGM(1,1) does not require input attributes determination and data preprocessing. As a result, SAIGM lessens the reliance on modelling expertise from the position of expert systems.

The next section (Section 2) of this paper outlines the methodology's general principles; Section 3, presents the simulation performance produced using SAIGM(1,1); and Section 4 concludes the paper.

## Method

In order to be able to predict the evolution of a system, it is necessary to collect sufficient information on this system beforehand, because future processes are very often linked to previous observations [7]. However, it may occasionally happen that we come across a system for which we do not have enough information. Fortunately, there are some more or less complex tools that can remedy this situation. These include linear regression [7], extrapolation, genetic algorithms [8], Grey Models (GM) [9], and artificial intelligence [10]. GM stands out because it is able to generate very accurate forecasts with only four observations [11]. This is an undeniable advantage for many research fields and situations where there is a lack of data.

Unfortunately, univariate first-order grey models (GM(1,1)) only yield accurate forecasts if the data used has a homogeneous exponential pattern [12]. In practice (like in energy consumption forecasting [13], accident prevention [14], crop forecasting [15] etc.) such situations almost never occur. It is therefore necessary to establish a GM(1,1) that works with all types of data and produces very accurate forecasts.

In general, the notation GM(p,q) designates a grey model established from an ordinary differential equation (called grey) of order p and having q variables within it. The set of variables is denoted by the notation $X^{\left(0\right)}$ which containing k components noted $x_{1}^{\left(0\right)},\;x_{2}^{\left(0\right)},\ldots ,\;x_{k}^{\left(0\right)}$. The superscript (0) indicates that the variable is raw (meaning it has not yet undergone any transformation). Once $X^{\left(0\right)}$ undergoes a transformation (an accumulation of some of its components for example), it is denoted $X^{\left(1\right)}$ and the transformed components are denoted $x_{1}^{\left(1\right)},\;x_{2}^{\left(1\right)},\ldots ,\;x_{k}^{\left(1\right)}$. This being said, in the following sections, we start by exposing the failures of GM(1,1). Then, we propose a new model and its properties, before validating it with theoretical and practical cases.

### Flaws of the standard GM(1,1) and its extension

We start off by recalling and outlining the shortcomings of GM(1,1), in order to explain the observation that we have made about its non-performance and why it is not generalizable. From there, we describe how to develop an intelligent auto-adaptive GM that can automatically modify its settings to lower forecasting errors no matter the type of series employed. There are also descriptions of the modelling approach, model's features, and parameter calculation.

Definition 1 [16]**:**$X^{\left(0\right)}$ is the system's raw entry sequence, defined by $X^{\left(0\right)}=\left(x_{1}^{\left(0\right)},\;x_{2}^{\left(0\right)},\ldots ,\;x_{k}^{\left(0\right)}\right)$; $x_{q}^{\left(0\right)}\geq 0\;\forall q=1,2,\ldots ,k$. The first-generation accumulation (1-AGO) is $X^{\left(1\right)}=\left(x_{1}^{\left(1\right)},\;x_{2}^{\left(1\right)},\ldots ,\;x_{k}^{\left(1\right)}\right)$ and calculated with Eq. (1):

(1) $$x_{q}^{\left(1\right)}=\sum_{m=1}^{q}x_{m}^{\left(0\right)}$$

In order to extract the system's evolution rule, 1-AGO is essential since it enables the removal of any potentially disruptive oscillations from the system [16].

Definition 2 [16]**:** Assume that the definitions of $X^{\left(0\right)}$ and $X^{\left(1\right)}$ remain the same**.** A new sequence $Z^{\left(1\right)}=\left(z_{1}^{\left(1\right)},\;z_{2}^{\left(1\right)},\ldots ,\;z_{k}^{\left(1\right)}\right)$, denoted as mean sequence derived from subsequent terms (or background value), is presented below. $Z^{\left(1\right)}$ is calculated as in Eq. (2):

(2) $$z_{q}^{\left(1\right)}=\left(1-\mu \right)x_{q-1}^{\left(1\right)}+\mu x_{q}^{\left(1\right)}$$

where $\mu =\frac{1}{2}$, thus Eq. (2) can be rewritten as in Eq. (3):

(3) $$z_{q}^{\left(1\right)}=\mu \left(x_{q-1}^{\left(1\right)}+x_{q}^{\left(1\right)}\right)$$

Definition 3 [16]**:** According to Definitions 1 and 2, Eqs. (4) and (5) below are referred to as the grey differential equation and the traditional GM(1,1) image's equation, respectively:

(4) $$x_{q}^{\left(0\right)}+\alpha z_{q}^{\left(1\right)}=\beta$$

(5) $$\frac{dx^{\left(1\right)}\left(t\right)}{dt}=-\alpha x^{\left(1\right)}\left(t\right)+\beta$$

Actually, GM(1,1) basic version is given by Eq. (4).

(−α) represents the development coefficient of ${\hat{x}}^{\left(1\right)}$ and ${\hat{x}}^{\left(0\right)}$, whereas β represents the grey action quantity. In general, the input variables of a grey system are external to it or must be predefined. Given that GM(1,1) is implemented with one type of sequence at a time, it uses for this purpose the sequence $Z^{\left(1\right)}$, disregarding any external sequences (called driving values). The parameter β is derived from $Z^{\left(1\right)}$ that translates the variations seen in the data into a greyed-out intention. This parameter represents the extension of the appropriate intention. Note that this feature distinguishes grey systems from black boxes.

Theorem 1 [16]***:****Using****Definitions 1****et****2****, we can calculate the vector*$\hat{a}=\left(\alpha ,\beta \right)$*by ordinary least squares*[17] (Eq. (6))*:*

(6) $$\hat{a}=\left[\begin{array}{c} \alpha \\ \beta \end{array}\right]={\left(B^{T}B\right)}^{-1}B^{T}Y$$

Where: $B=\left(\begin{array}{c} \begin{array}{c} -z_{2}^{\left(1\right)}\\ -z_{3}^{\left(1\right)}\\ \vdots \end{array}\\ -z_{k}^{\left(1\right)} \end{array}\;\begin{array}{c} \begin{array}{c} 1\\ 1\\ \vdots \end{array}\\ 1 \end{array}\;\right)\in R^{(k-1)\;\times \;2}$; $Y=\left(\begin{array}{c} \begin{array}{c} x_{2}^{\left(0\right)}\\ x_{3}^{\left(0\right)}\\ \vdots \end{array}\\ x_{k}^{\left(0\right)} \end{array}\right)\;\in R^{(k-1)\;\times \;1}$

Theorem 2 [16]: *Suppose the matrices*$\hat{a},\;Y$*and*B*are still the same as those in*Theorem 1*, then,*Eqs. (7), (8)*and*(9)*are called the time response function, the time response sequence, and the restored values respectively*.

(7) $$x^{\left(1\right)}\left(t\right)=\left(x_{1}^{\left(1\right)}-\frac{\beta }{\alpha }\right)e^{-\alpha t}+\frac{\beta }{\alpha }$$

${\hat{x}}_{t=1}^{\left(1\right)}=x_{1}^{\left(1\right)}$ is the initial condition for the solution of the image equation.

(8) $${\hat{x}}_{q+1}^{\left(1\right)}=\left(x_{1}^{\left(1\right)}-\frac{\beta }{\alpha }\right)e^{-\alpha q}+\frac{\beta }{\alpha },\;q=1,2,\ldots ,\;k$$

(9) $${\hat{x}}_{q+1}^{\left(0\right)}={\hat{x}}_{q+1}^{\left(1\right)}-{\hat{x}}_{q}^{\left(1\right)}=\left(1-e^{\alpha }\right)\left(x_{1}^{\left(1\right)}-\frac{\beta }{\alpha }\right)e^{-\alpha q}$$

If $\left(1-e^{\alpha }\right)\left(x_{1}^{\left(1\right)}-\frac{\beta }{\alpha }\right)=\lambda$, the following homogeneous exponential function is obtained:

(10) $${\hat{x}}_{q+1}^{\left(0\right)}=\lambda e^{-\alpha q}$$

Eq. (10) allows us to make two observations when we implement GM(1,1) with the sequence $X^{\left(0\right)}=\left(x_{1}^{\left(0\right)},\;x_{2}^{\left(0\right)},\ldots ,\;x_{k}^{\left(0\right)}\right)$ to obtain the predictive simulations of the sequence ${\hat{X}}^{\left(0\right)}=\left\{{\hat{x}}_{1}^{\left(0\right)},\;{\hat{x}}_{2}^{\left(0\right)},\ldots ,\;{\hat{x}}_{k}^{\left(0\right)}\right\}$:

i If $X^{\left(0\right)}$ is an exponential heterogeneous series rather than a homogeneous exponential rule, ${\hat{X}}^{\left(0\right)}$ produces forecasts that deviate greatly from actual data. The reason is that ${\hat{X}}^{\left(0\right)}$ will adhere to a pure exponential law unlike $X^{\left(0\right)}$ from which ${\hat{X}}^{\left(0\right)}$ was derived.
ii If $X^{\left(0\right)}$ is an exponential homogeneous series, then assuming that $x_{q}^{\left(0\right)}=\lambda_{1}e^{-\alpha (q-1)},q=1,2,\ldots ,k\;$; we quickly observe that the terms of the forecasting sequence ${\hat{X}}^{\left(0\right)}$ derived from Eq. (10) are all distinct, because $\lambda_{1}\neq \lambda$.

The preceding analysis may lead us to believe that the standard GM(1,1) performs best with exponentially heterogeneous series. The fixed structure and parametrization of GM lead to this restriction. In addition, the second analysis concludes that it will still produce unreliable results even if the series obey the exponential homogeneous law. Therefore, the standard GM(1,1) model cannot adequately extract the law of evolution of a generalized system (real-world) as a result in both instances.

Additionally, even enhanced variants of GM(1,1) are unsuccessful [18]. Only a particular kind of series can be used to implement these versions. Therefore, it is essential to create a strategy to close this gap.

Definition 4 $X^{\left(0\right)}$, $X^{\left(1\right)}$ and $Z^{\left(1\right)}$ still have the same previous definitions and γ∈R. Also, q is still defined as in Definition 1. Then, Eq. (11) is the extended form of the traditional GM(1,1):

(11) $$x_{q}^{\left(0\right)}+\alpha z_{q}^{\left(1\right)}=q\beta +\gamma$$

Recall that $x_{q}^{\left(0\right)}=x_{q}^{\left(1\right)}-x_{q-1}^{\left(1\right)}$. Thus, we can infer from Definitions 2 and 4 that Eq. (11) leads to Eq. (12):

(12) $$\left(1+\mu \alpha \right)x_{q}^{\left(1\right)}=\left(1-\mu \alpha \right)x_{q-1}^{\left(1\right)}+q\beta +\gamma$$

(13) $$\Rightarrow \;x_{q}^{\left(1\right)}=\omega_{1}x_{q-1}^{\left(1\right)}+q\omega_{2}+\omega_{3}$$

Where : $\omega_{1}=\frac{1-\mu \alpha }{1+\mu \alpha };\;\omega_{2}=\;\frac{\beta }{1+\mu \alpha }$ et $\omega_{3}=\frac{\gamma }{1+\mu \alpha }$

The temporal response function of $X^{\left(1\right)}$ is expressed in Eq. (13).

### SAIGM(1,1) parameterization

The calculation of $\omega_{1}$, $\omega_{2}$ and $\omega_{3}$ in this paper uses ordinary least squares and linear algebraic formulas. The values of α, β and γ and are then deduced from $\omega_{1}$, $\omega_{2}$ and $\omega_{3}$. The disparities between actual $x^{\left(1\right)}$ and predicted values ${\hat{x}}^{\left(1\right)}$ during simulations must be kept to a minimal Δ as follows:

$$\Delta =\text{min}\sum_{q=2}^{k}{\left(x_{q}^{\left(1\right)}-{\hat{x}}_{q}^{\left(1\right)}\right)}^{2}$$

and Eq. (13) shows that Δ results in:

$$\Delta =\text{min}\sum_{q=2}^{k}{\left(x_{q}^{\left(1\right)}-\omega_{1}{\hat{x}}_{q-1}^{\left(1\right)}-q\omega_{2}-\omega_{3}\right)}^{2}$$

Ordinary least squares is used to minimize Δ in relation to $\omega_{1}$, $\omega_{2}$ and $\omega_{3}$. The resulting system (S) is as follows:

$$\left(S\right):\left\{ \begin{array}{c} \partial_{\omega_{1}}\Delta =-2\sum_{q=2}^{k}{\hat{x}}_{q-1}^{\left(1\right)}\left(x_{q}^{\left(1\right)}-\omega_{1}{\hat{x}}_{q-1}^{\left(1\right)}-q\omega_{2}-\omega_{3}\right)=0\\ \partial_{\omega_{2}}\Delta =-2\sum_{q=2}^{k}q\left(x_{q}^{\left(1\right)}-\omega_{1}{\hat{x}}_{q-1}^{\left(1\right)}-q\omega_{2}-\omega_{3}\right)=0\;\\ \partial_{\omega_{3}}\Delta =-2\sum_{q=2}^{k}\left(x_{q}^{\left(1\right)}-\omega_{1}{\hat{x}}_{q-1}^{\left(1\right)}-q\omega_{2}-\omega_{3}\right)=0\; \end{array}\right.$$

The terms in (S) are rearranged to give than is Eq. (14):

(14) $$\left(S\right):\left\{ \begin{array}{c} \omega_{1}\sum_{q=2}^{k}{\left({\hat{x}}_{q-1}^{\left(1\right)}\right)}^{2}+\omega_{2}\sum_{q=2}^{k}q{\hat{x}}_{q-1}^{\left(1\right)}+\omega_{3}\sum_{q=2}^{k}{\hat{x}}_{q-1}^{\left(1\right)}=\sum_{q=2}^{k}{\hat{x}}_{q-1}^{\left(1\right)}x_{q}^{\left(1\right)}\\ \omega_{1}\sum_{q=2}^{k}q{\hat{x}}_{q-1}^{\left(1\right)}+\omega_{2}\sum_{q=2}^{k}q^{2}+\omega_{3}\sum_{q=2}^{k}q=\sum_{q=2}^{k}qx_{q}^{\left(1\right)}\;\\ \omega_{1}\sum_{q=2}^{k}{\hat{x}}_{q-1}^{\left(1\right)}+\omega_{2}\sum_{q=2}^{k}q+\left(k-1\right)\omega_{3}=\sum_{q=2}^{k}x_{q}^{\left(1\right)}\; \end{array}\right.$$

The system (S) can be rewritten as MΩ=Ψ, where:

$M=\left(\begin{array}{ccc} \sum_{q=2}^{k}{\left({\hat{x}}_{q-1}^{\left(1\right)}\right)}^{2} & \sum_{q=2}^{k}q{\hat{x}}_{q-1}^{\left(1\right)} & \sum_{q=2}^{k}{\hat{x}}_{q-1}^{\left(1\right)}\\ \sum_{q=2}^{k}q{\hat{x}}_{q-1}^{\left(1\right)} & \sum_{q=2}^{k}q^{2} & \sum_{q=2}^{k}q\\ \sum_{q=2}^{k}{\hat{x}}_{q-1}^{\left(1\right)} & \sum_{q=2}^{k}q & \left(k-1\right) \end{array}\right)\in R^{3\;\times \;3}$; $\Psi =\left(\begin{array}{c} \sum_{q=2}^{k}{\hat{x}}_{q-1}^{\left(1\right)}x_{q}^{\left(1\right)}\\ \sum_{q=2}^{k}qx_{q}^{\left(1\right)}\\ \sum_{q=2}^{k}x_{q}^{\left(1\right)} \end{array}\right)\in R^{3\;\times \;1}$ and $\Omega =\left(\begin{array}{c} \omega_{1}\\ \omega_{2}\\ \omega_{3} \end{array}\right)\in R^{3\;\times \;1}$

The system of equations (S) is linear with a unique solution. Hence, it is possible to apply Cramer's rule to calculate $\omega_{1}$, $\omega_{2}$ and $\omega_{3}$, and deduce α,β, and γ from the calculations [19]. Consequently, if detM represents the determinant of M, while $detM_{\omega_{1}}$, $detM_{\omega_{2}}$, and $detM_{\omega_{3}}$ denote the determinants of numerators of solutions $\omega_{1}$, $\omega_{2}$ and $\omega_{3}$, respectively, therefore:

$$\left\{ \begin{array}{c} \omega_{1}=\frac{detM_{\omega_{1}}}{detM}=\frac{1-\mu \alpha }{1+\mu \alpha }\\ \omega_{2}=\frac{detM_{\omega_{2}}}{detM}=\frac{\beta }{1+\mu \alpha }\\ \omega_{3}=\frac{detM_{\omega_{3}}}{detM}=\frac{\gamma }{1+\mu \alpha } \end{array}\right.\;\Rightarrow \;\left\{ \begin{array}{c} \alpha =-2+\frac{4}{1+\omega_{1}}\\ \beta =\frac{2\omega_{2}}{1+\omega_{1}}\;\\ \gamma =\frac{2\omega_{3}}{1+\omega_{1}}\; \end{array}\right.$$

The predicted values for ${\hat{x}}_{q}^{\left(1\right)}$ (Eq. (15)) are calculated by substituting α, β and γ in Eq. (13).

(15) $${\hat{x}}_{q}^{\left(1\right)}=\frac{1-\mu \alpha }{1+\mu \alpha }{\hat{x}}_{q-1}^{\left(1\right)}+\frac{q\beta +\gamma }{1+\mu \alpha }$$

### Modelling procedure of SAIGM(1,1)

We have thus far seen how to arrive at ${\hat{x}}_{q}^{\left(1\right)}$. These forecasts, however, represent the sums of the initial sequences ${\hat{x}}_{q}^{\left(0\right)}$. The restored value of ${\hat{x}}_{q}^{\left(1\right)}$ is ${\hat{x}}_{q}^{\left(0\right)}$ in accordance with Theorem 2, such that:

(16) $${\hat{x}}_{q}^{\left(0\right)}={\hat{x}}_{q}^{\left(1\right)}-{\hat{x}}_{q-1}^{\left(1\right)}$$

We obtain Eqs. (17) and (18) when q is given respective values of 2 and 3 in Eq. (15):

(17) $${\hat{x}}_{2}^{\left(1\right)}=\frac{1-\mu \alpha }{1+\mu \alpha }{\hat{x}}_{1}^{\left(1\right)}+\frac{2\beta +\gamma }{1+\mu \alpha }$$

(18) $${\hat{x}}_{3}^{\left(1\right)}=\frac{1-\mu \alpha }{1+\mu \alpha }{\hat{x}}_{2}^{\left(1\right)}+\frac{3\beta +\gamma }{1+\mu \alpha }$$

Eq. (17) is substituted into Eq. (18) to produce,

(19) $${\hat{x}}_{3}^{\left(1\right)}={\left(\frac{1-\mu \alpha }{1+\mu \alpha }\right)}^{2}{\hat{x}}_{1}^{\left(1\right)}+\frac{1-\mu \alpha }{1+\mu \alpha }\left(\frac{2\beta +\gamma }{1+\mu \alpha }\right)+\frac{3\beta +\gamma }{1+\mu \alpha }$$

Correspondingly, we obtain Eq. (20) when q in Eq. (15) is given the value 4,

(20) $${\hat{x}}_{4}^{\left(1\right)}=\frac{1-\mu \alpha }{1+\mu \alpha }{\hat{x}}_{3}^{\left(1\right)}+\frac{4\beta +\gamma }{1+\mu \alpha }$$

${\hat{x}}_{3}^{\left(1\right)}$ is then substituted in Eq. (20) to give Eq. (21),

(21) $${\hat{x}}_{4}^{\left(1\right)}={\left(\frac{1-\mu \alpha }{1+\mu \alpha }\right)}^{3}{\hat{x}}_{1}^{\left(1\right)}+{\left(\frac{1-\mu \alpha }{1+\mu \alpha }\right)}^{2}\left(\frac{2\beta +\gamma }{1+\mu \alpha }\right)+\frac{1-\mu \alpha }{1+\mu \alpha }\left(\frac{3\beta +\gamma }{1+\mu \alpha }\right)+\frac{4\beta +\gamma }{1+\mu \alpha }$$

Therefore, for ${\hat{x}}_{j}^{\left(1\right)}$, with,j=2,3,…; we get,

(22) $${\hat{x}}_{j}^{\left(1\right)}=\frac{1-\mu \alpha }{1+\mu \alpha }{\hat{x}}_{j-1}^{\left(1\right)}+\frac{j\beta +\gamma }{1+\mu \alpha }$$

Eq. (22) is expressed as in Eq. (23), when expanded.

(23) $${\hat{x}}_{j}^{\left(1\right)}\;=\;{\left(\frac{1-\mu \alpha }{1+\mu \alpha }\right)}^{j-1}{\hat{x}}_{1}^{\left(1\right)}+\left(\frac{2\beta +\gamma }{1+\mu \alpha }\right){\left(\frac{1-\mu \alpha }{1+\mu \alpha }\right)}^{j-2}+\left(\frac{3\beta +\gamma }{1+\mu \alpha }\right){\left(\frac{1-\mu \alpha }{1+\mu \alpha }\right)}^{j-3}+\ldots +\frac{1-\mu \alpha }{1+\mu \alpha }\left(\frac{\left(j-1\right)\beta +\gamma }{1+\mu \alpha }\right)+\frac{j\beta +\gamma }{1+\mu \alpha }{\left(\frac{1-\mu \alpha }{1+\mu \alpha }\right)}^{0}$$

And Eq. (23) is easily condensed as,

(24) $${\hat{x}}_{j}^{\left(1\right)}={\left(\frac{1-\mu \alpha }{1+\mu \alpha }\right)}^{j-1}{\hat{x}}_{1}^{\left(1\right)}+\sum_{h=0}^{j-2}\left\{\left(\frac{\left(j-h\right)\beta +\gamma }{1+\mu \alpha }\right){\left(\frac{1-\mu \alpha }{1+\mu \alpha }\right)}^{h}\right\}$$

Given that Eq. (16) states that ${\hat{x}}_{q}^{\left(0\right)}={\hat{x}}_{q}^{\left(1\right)}-{\hat{x}}_{q-1}^{\left(1\right)}$, the expression for ${\hat{x}}_{q}^{\left(0\right)}$ with the help of Eq. (24) is,

(25) $${\hat{x}}_{q}^{\left(0\right)}={\left(\frac{1-\mu \alpha }{1+\mu \alpha }\right)}^{q-1}{\hat{x}}_{1}^{\left(1\right)}+\sum_{h=0}^{q-2}\left\{\left(\frac{\left(q-h\right)\beta +\gamma }{1+\mu \alpha }\right){\left(\frac{1-\mu \alpha }{1+\mu \alpha }\right)}^{h}\right\}-{\left(\frac{1-\mu \alpha }{1+\mu \alpha }\right)}^{q-2}{\hat{x}}_{1}^{\left(1\right)}-\sum_{h=0}^{q-3}\left\{\left(\frac{\left(q-h-1\right)\beta +\gamma }{1+\mu \alpha }\right){\left(\frac{1-\mu \alpha }{1+\mu \alpha }\right)}^{h}\right\}$$

The terms in Eq. (25) are rearranged to produce the expression shown in Eq. (26).

(26) $${\hat{x}}_{q}^{\left(0\right)}=\left\{\left(\frac{-\alpha }{1+\mu \alpha }{\hat{x}}_{1}^{\left(0\right)}+\frac{2\beta +\gamma }{1+\mu \alpha }\right){\left(\frac{1-\mu \alpha }{1+\mu \alpha }\right)}^{q-2}\right\}+\sum_{h=0}^{q-3}\frac{\beta }{1+\mu \alpha }{\left(\frac{1-\mu \alpha }{1+\mu \alpha }\right)}^{h}$$

If we let $(\frac{-\alpha }{1+\mu \alpha }{\hat{x}}_{1}^{\left(0\right)}+\frac{2\beta +\gamma }{1+\mu \alpha })=\Lambda$, this allows for the reduction of ${\hat{x}}_{q}^{\left(0\right)}$ to,

(27) $${\hat{x}}_{q}^{\left(0\right)}=\Lambda \omega_{1}^{\left(q-2\right)}+\sum_{h=0}^{q-3}\omega_{2}\omega_{1}^{h},\;q\geq 2$$

**Fig. 1:**
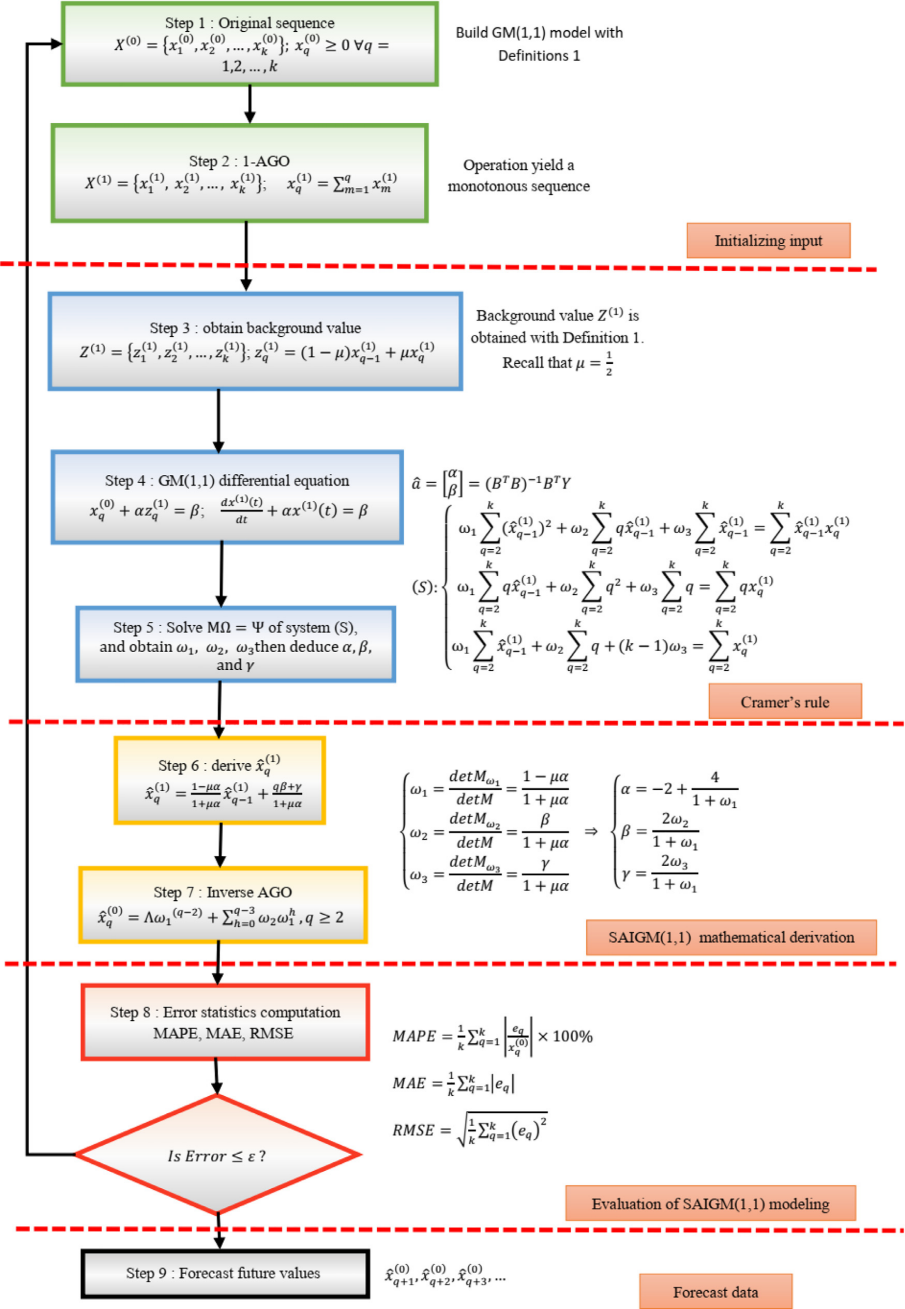
Flowchart of SAIGM(1,1) model.

Structural intelligent auto-adaptive grey model (SAIGM) is the name given to Eq. (27). The SAIGM flowchart is shown in Fig. 1.

## Evaluation of the proposed SAIGM(1,1) model

### Comparing the success rates of predictions with ideal data

We consider SADGM [5], and TDPDGM [20] which are amongst recent intelligent GMs to compare SAIGM's performances on their capacity to predict homogeneous exponential series $\left(X_{1}\right)$, heterogeneous exponential series $\left(X_{2}\right)$, quasi heterogeneous exponential series $\left(X_{3}\right)$, random series $\left(X_{4}\right)$, and linear series $\left(X_{5}\right)$ as in [21].

$$X_{1}:x_{q}^{\left(0\right)}=0.8\cdot 1.5^{q},\;q=1,2,\ldots ,20$$

$$X_{2}:x_{q}^{\left(0\right)}=1.3\cdot 1.8^{q}+3.5,\;q=1,2,\ldots ,20$$

$$X_{3}:x_{q}^{\left(0\right)}\approx 1.3\cdot 1.8^{q}+1.6,\;q=1,2,\ldots ,20$$

$$\begin{array}{ccc} & & X_{4}:\;\{73.5484,\;45.2685,\;75.6498,\;65.1462,\;89.3647,\;58.0210,\;36.2646,\;56.8974,\;98.3254,\;32.0175,\;88.2019,\;58.2522,\\ & & \;83.0855,\;22.3649,\;47.9024,\;71.2643,\;60.4652,\;25.4500,\;37.0207,\;43.0877\} \end{array}$$

$$X_{5}:x_{q}^{\left(0\right)}=2.4q+3.5,\;q=1,2,\ldots ,20$$

In the event of data leaking, overfitting is a possibility. To make sure that the models are neither underfitting nor overfitting, the dataset is split into training (or modelling) set and test (or validation) set. This prevents data leaking. Thus, by splitting the data into two, the models are able to assess the generalizability on data that were hidden during the modelling stage.

The first 15 data points (q=1,2,…,15) are used for training, while the remaining 5 data points (q=16,17,…,20) are used for testing. To calculate the error $e_{q}$ between actual $x_{q}^{\left(0\right)}$ and predicted ${\hat{x}}_{q}^{\left(0\right)}$ data, three evaluation criteria are used: root mean square error (RMSE) (Eq. (28)), mean absolute error (MAE) (Eq. (29)), and mean absolute percentage error (MAPE) (Eq. (30)). The best model that with the error statistics closest to 0.

(28) $$\sqrt{\frac{1}{k}\sum_{q=1}^{k}{\left(e_{q}\right)}^{2}}$$

(29) $$\frac{1}{k}\sum_{q=1}^{k}\left|e_{q}\right|$$

(30) $$\frac{1}{k}\sum_{q=1}^{k}\left|\frac{e_{q}}{x_{q}^{\left(0\right)}}\right|\times \;100\%$$

**Fig. 2:**
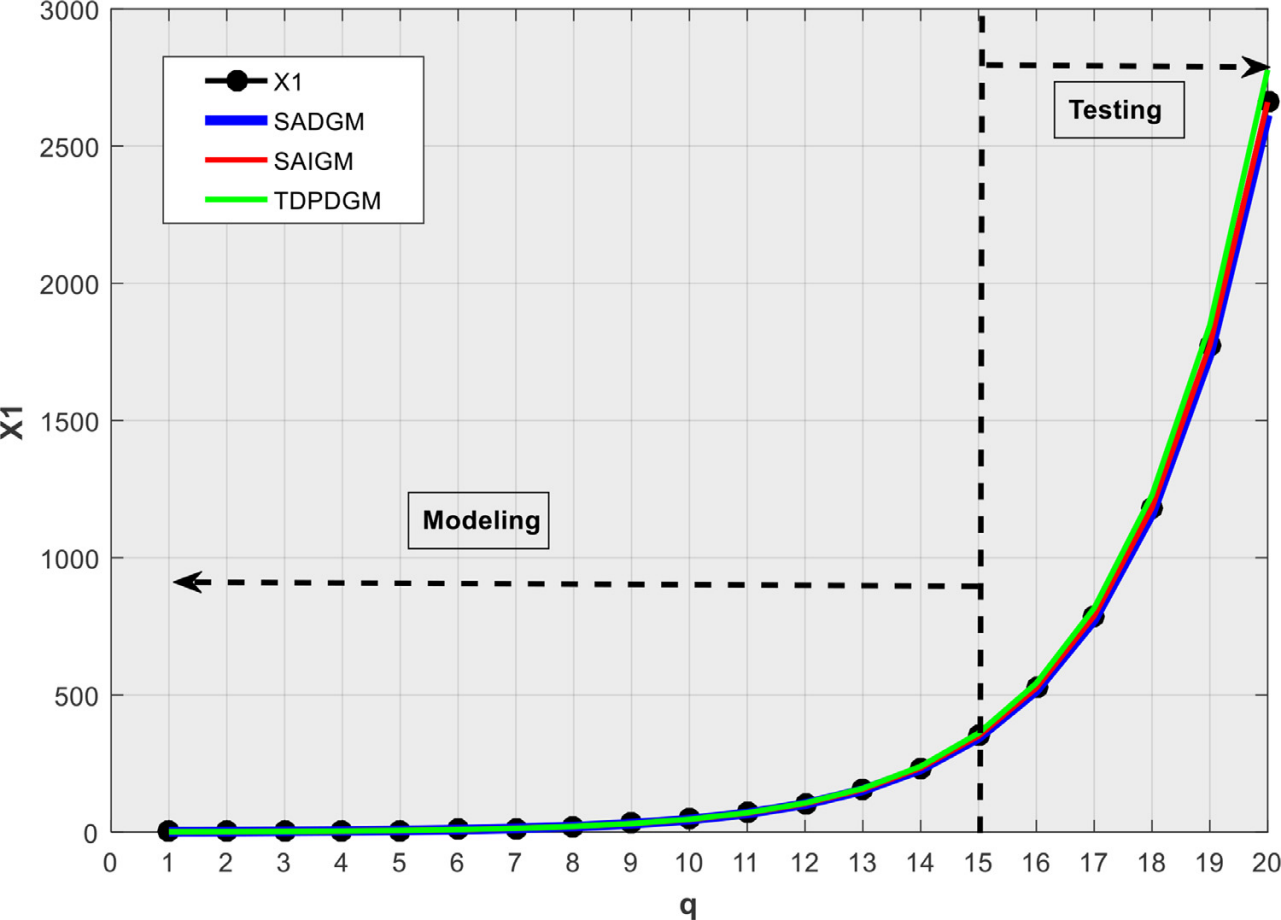
Verification using homogenous exponential sequence ($X_{1}$).

**Fig. 3:**
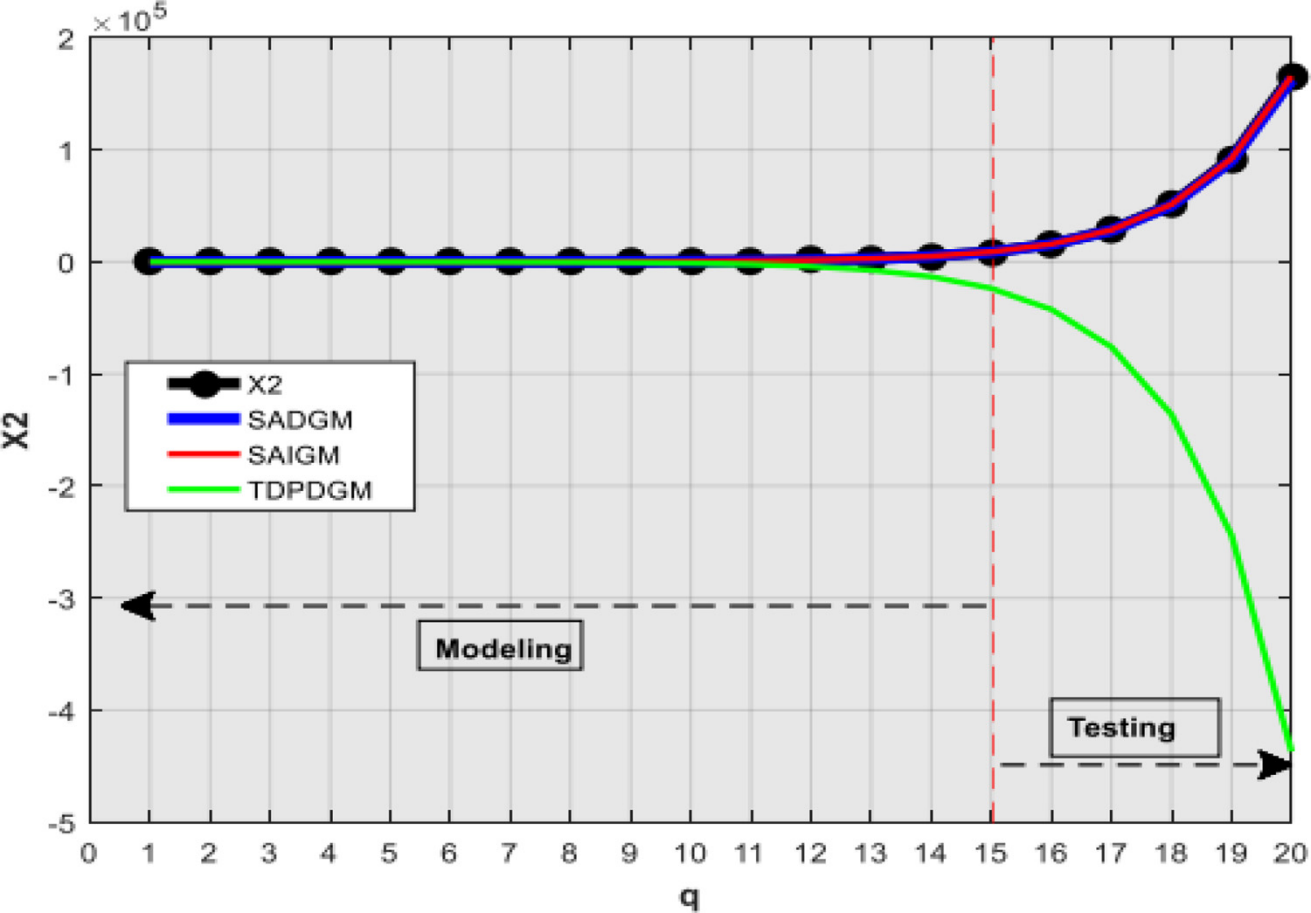
Verification using heterogeneous exponential sequence ($X_{2}$).

**Fig .4:**
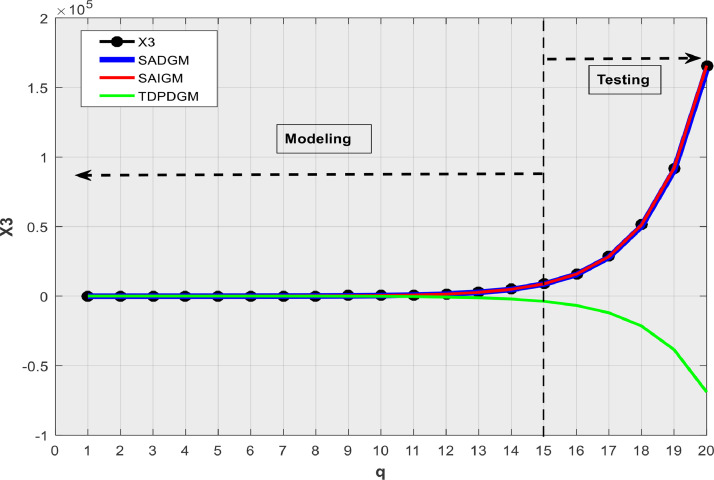
Verification using approximate heterogeneous exponential sequence ($X_{3}$).

**Fig. 5:**
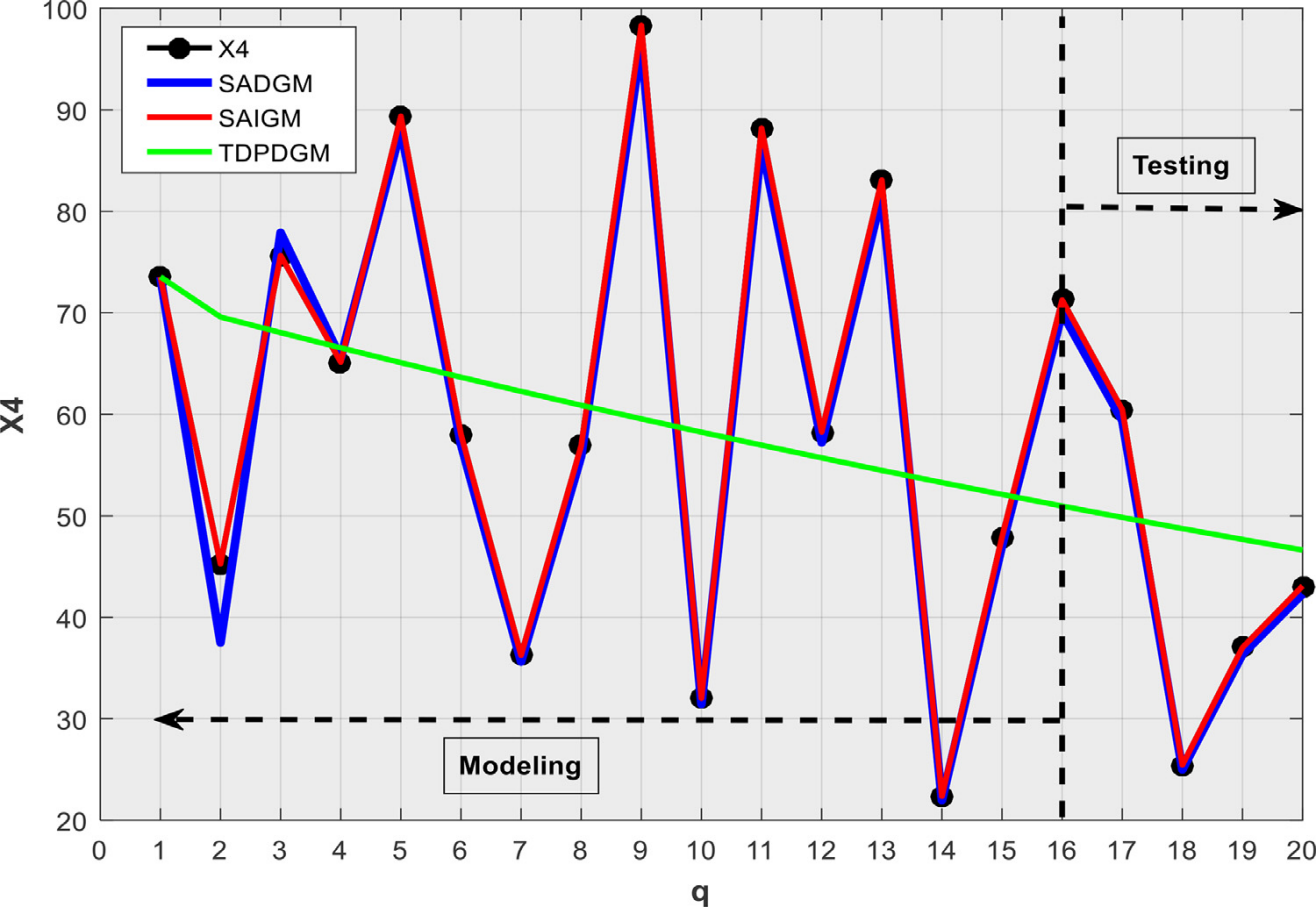
Verification using random sequence ($X_{4}$).

**Fig. 6:**
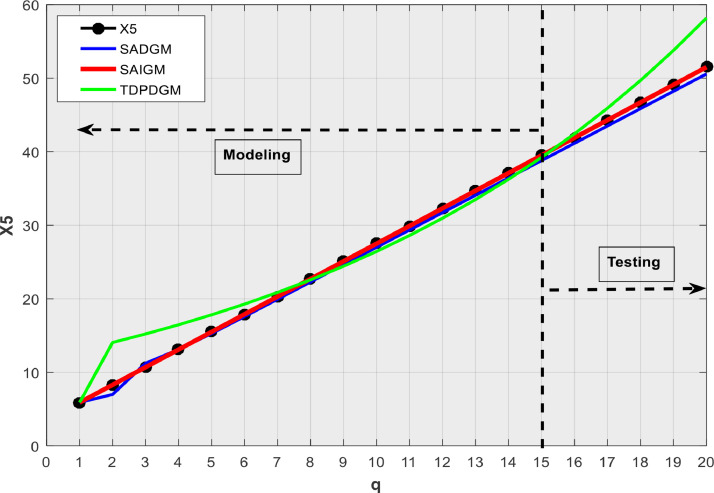
Verification using linear sequence ($X_{5}$).

Table 1 presents the simulation outcomes, while Fig. 2, Fig. 3, Fig .4, Fig. 5, Fig. 6 show the forecasts in graphical form. We note that no matter the system to which SAIGM is applied, it produces unbiased forecasts. SAIGM can effectively excavate the trend and evolution of the grey system to which it is applied, regardless of whether the system's series are homogenous exponential (Fig. 2), heterogeneous exponential (Fig. 3), approximate heterogeneous exponential (Fig. 4), random (Fig. 5), or linear (Fig. 6).

**Table 1:** Performance of intelligent GMs on various types of series.

Model	Data	RMSE					MAE					MAPE				
		$X_{1}$	$X_{2}$	$X_{3}$	$X_{4}$	$X_{5}$	$X_{1}$	$X_{2}$	$X_{3}$	$X_{4}$	$X_{5}$	$X_{1}$	$X_{2}$	$X_{3}$	$X_{4}$	$X_{5}$
SADGM	Training	32.19	49.06	29.03	12.31	10.57	14.78	14.06	14.04	15.33	10.32	9.1	4.4	2.61	7.2	5.0
	Validation	91.8	82.409	12.409	15.14	13.52	13.81	15.8	15.8	16.42	12.36	3.7	2.3	2.27	8.30	4.99
SAIGM	Training	1.74	7.11	2.71	4.54	1.21	2.9	5.0	7.1	2.0	1.58	1.26	2.66	1.2	2.67	0
	Validation	5.93	2.71	1.81	9.15	5.51	3.4	7.5	3.1	8.30	3.09	1.23	2.27	2.2	2.1	0
TDPDDGM	Training	50.98	58.90	23.52	21.4	11.85	21.67	23.5	25.56	45.79	23.42	2.63	195.4	80.74	35.68	8.13
	Validation	74.02	29.73	29.73	10.37	10.15	26.2	28.8	28.8	30.76	20.02	4.3	4.11	4.11	22.8	9.1

SAIGM's flexible structure helps to take into account the quirks of data from a generalized system. Competing intelligent GMs can only be applied to systems with one characteristic at a time since their structural flexibility is insufficient. This explains why TDPDGM deviates from actual data in Fig. 3, Fig .4, Fig. 5, Fig. 6. However, in reality, real-world systems never show just one trait, but rather a combination of them. Competing intelligent GMs like SADGM cannot be precise for such systems. Given all this, we decided to check the performance of the new model with data from a real-world system.

### Comparing the success rates of predictions with real-world data

**Fig. 7:**
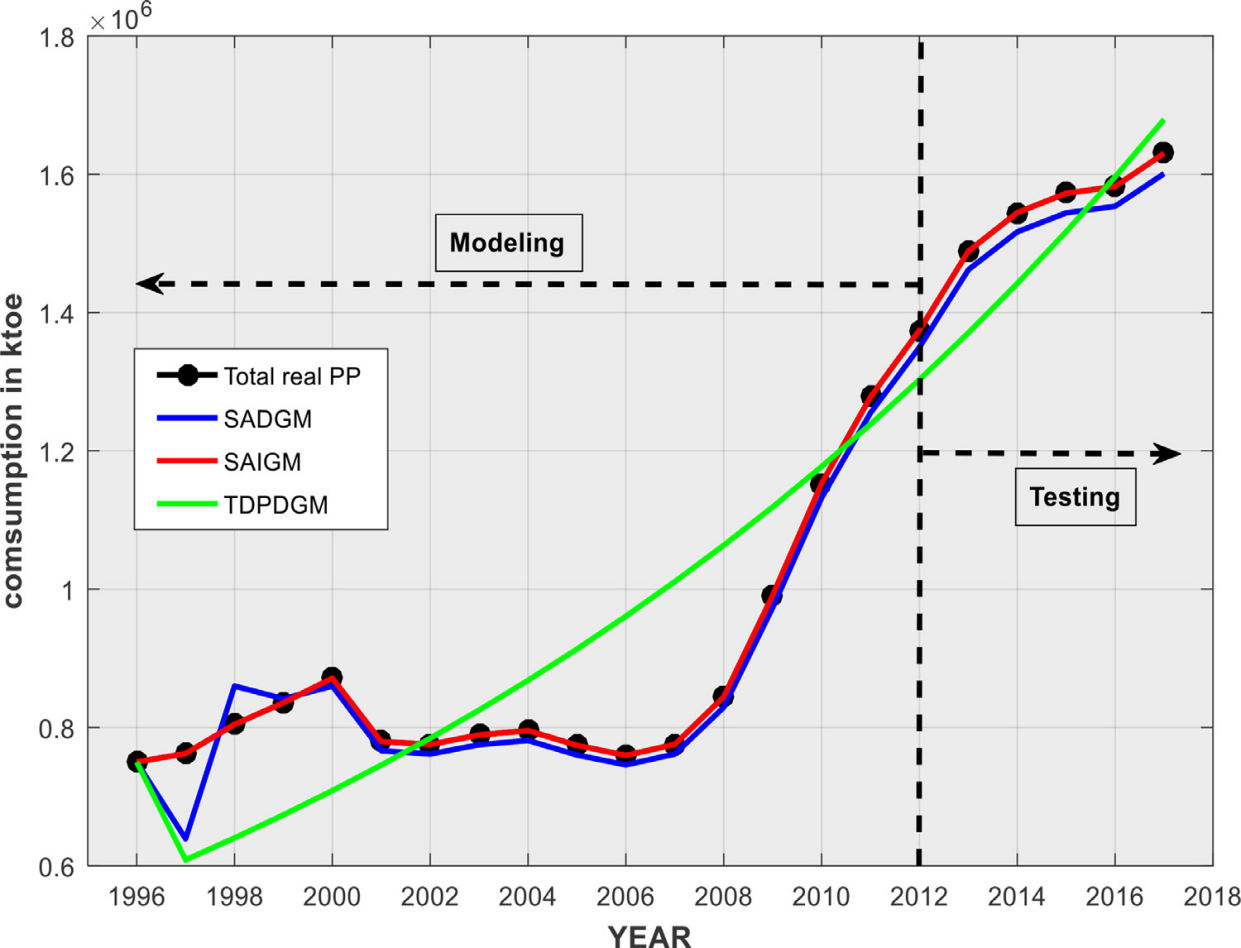
Verification of prediction fit curves using real PP consumption data.

Cameroun petroleum products (PP) consumption data for the years 1996–2012 are used to implement TDPDGM, SADGM, and SAIGM and simulate predictions for the years 2013–2017. Table 2 provides statistical forecast errors for the aforementioned intelligent GMs, while Fig. 7 displays the predictions fit curves.

**Table 2:** Performance of intelligent GMs when applied to PP demand prediction.

Model		RMSE	MAE	MAPE
SADGM	Training	15.85	13.9	2.74
	Validation	12.50	15.10	5.10
SAIGM	Training	1.09	1.10	1.40
	Validation	3.10	9.80	1.54
TDPDGM	Training	72.52	24.10	11.59
	Validation	42.16	32.70	10.30

**Fig. 8:**
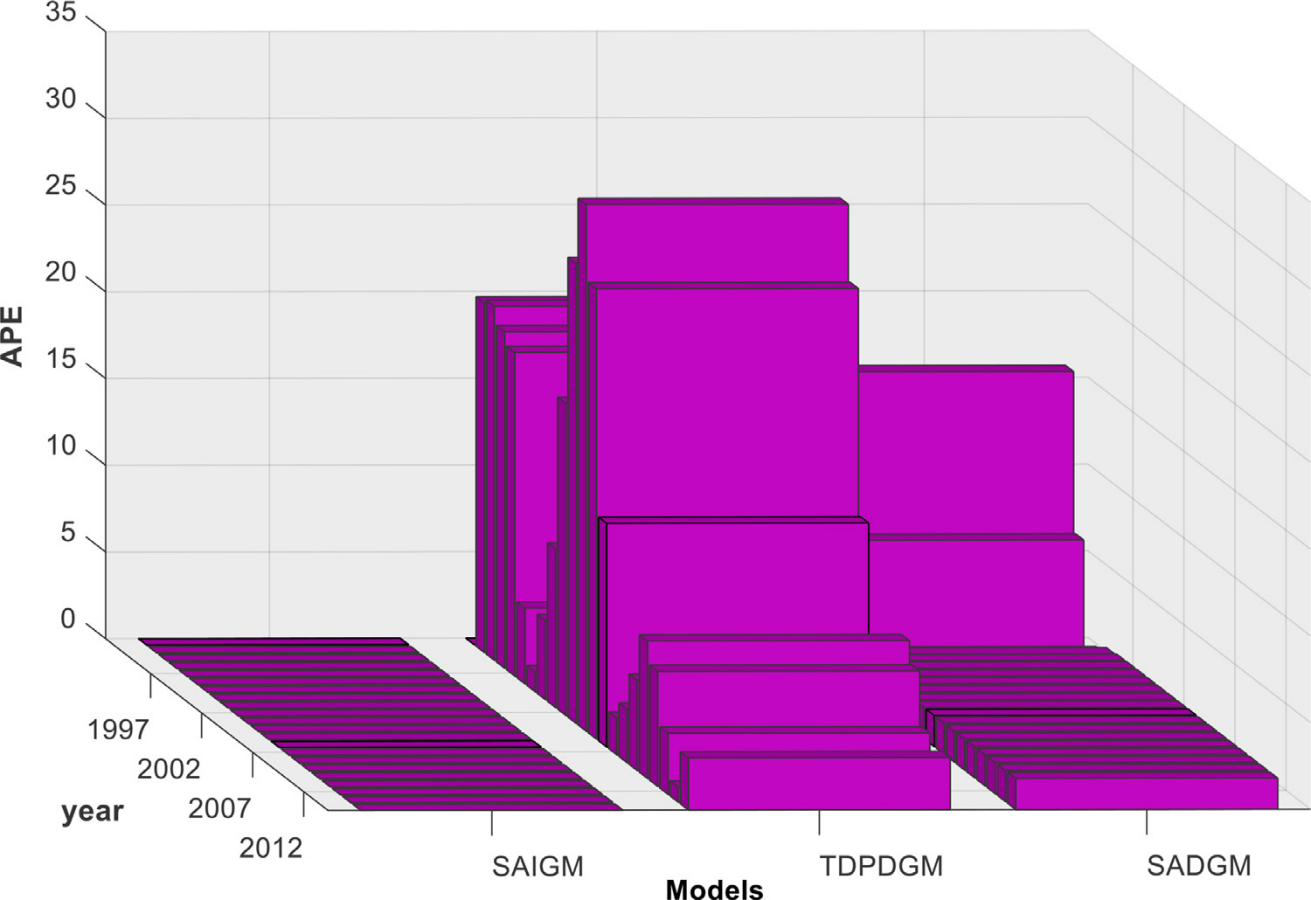
APE distributions of SADGM, SAIGM and TDPDGM.

Fig. 7 shows that SADGM's forecasts are obviously below the system's actual evolution trend. The forecasting curves demonstrate that SAIGM performs noticeably better than TDPDGM and SADGM. More significantly, despite the fact that SADGM necessitates a brief training phase prior to modelling, SAIGM and SADGM can capture both the system's evolution and trend in comparison to TDPDGM. The latter's forecasts rather oscillate between the two sides of the real data without settling on either of them. The absolute percentage errors (APEs) shown in Fig. 8 and performance metrics (given by Eqs. (28-30)) of SAIGM are significantly lower than those of TDPDGM and SADGM. These error statistics attest SAIGM's forecasting abilities and its capacity to excavate the evolution law of a real-world system.

It should be noted, however, that the mathematical formulation of the TDPDGM indicates that it is not appropriate to use it with all types of series. More so, TDPDGM is a discrete model and its time response function (given by Eq. (31)) combines an exponential function, a discrete integral, an exponential function and the polynomial function f(t) (represented by Eq. (32)) (see Ma and Liu [20], pp. 19).

(31) $${\hat{x}}_{1}^{\left(1\right)}\left(t\right)=x_{1}^{\left(0\right)}\left(1\right)e^{a\left(1-t\right)}+\sum_{\tau =2}^{t}\frac{1}{2}\left(f\left(\tau \right)e^{a\left(\tau -t\right)}+f\left(\tau -1\right)e^{a\left(\tau -t-1\right)}\right)$$

(32) $$f\left(t\right)=b\sum_{\tau =1}^{t}\tau^{2}+c\sum_{\tau =1}^{t}\tau +d$$

Therefore, the TDPDGM model is not able to yield precise forecasts when implemented with series that do not approximately fit Eqs. (31) and (32). The TDPDGM model, unlike its counterparts, has a mathematical formulation that is not flexible when implemented with input data that deviates from that for which it was designed. This proves that a preliminary study should be carried out to determine the nature of the input data before choosing which model is the most appropriate, except for the new SAIGM that this paper proposes.

## Conclusion

An intelligent auto-adaptive grey structural forecasting model (SAIGM) that can be used with both ideal and generalized series is developed in this paper. An experimental verification using the predicted PP demand for Cameroon is carried out. The innovative SAIGM model has the following benefits over earlier intelligent models that have been published in the literature but suffer from inflexible framework and inadequate adaption:

• SAIGM's structure is much more flexible since it automatically enhances and intelligently adjusts the parameters of the conventional GM(1,1).
• SAIGM's can define the intrinsic structural model on its own and adjust it to the real properties of the modelling data, regardless of whether the data is random, linear, heterogeneous exponential, homogeneous exponential or even a mix of any of them.
• The addition of a cumulative generation operator eliminates any potential disturbance in the series. Additionally, the time response function strengthens the system's evolution law, allows for its extraction, and minimizes errors caused by information loss. By ensuring simulation stability in this way, predictions are improved. In light of this, SAIGM reduces the need for modelling knowledge from the context of expert systems.

Overall, the SAIGM model presented in this paper has a high level of prediction accuracy, adaptability, feasibility, and generalizability, and demonstrates that there is still margin of progress for GM(1,1) optimization.

## Funding

This research did not receive any specific grant from funding agencies in the public, commercial, or not-for-profit sectors.

## CRediT authorship contribution statement

**Flavian Emmanuel Sapnken:** Conceptualization, Methodology, Data curation, Validation, Investigation, Software, Writing – original draft. **Jean Gaston Tamba:** Supervision, Visualization, Validation, Writing – review & editing.

## Declaration of Competing Interests

The authors declare that they have no known competing financial interests or personal relationships that could have appeared to influence the work reported in this paper.

## Data Availability

Data will be made available on request.
